# Development and validation of a nomogram for predicting the risk of preoperative deep vein thrombosis in patients with Pilon fractures: A retrospective cohort study

**DOI:** 10.1097/MD.0000000000047618

**Published:** 2026-02-13

**Authors:** Yongxuan Kang, Yang Zhang, Kai Kang, Tong Li, Xuebin Zhang, Chenni Ji

**Affiliations:** a Department of Orthopedic Surgery, The Third Hospital of Hebei Medical University, Shijiazhuang, Hebei, People’s Republic of China.

**Keywords:** deep vein thrombosis, incidence, nomogram, Pilon fractures, risk factors

## Abstract

This study aimed to identify risk factors for preoperative deep vein thrombosis (DVT) in Pilon fracture patients and develop a nomogram model. This study retrospectively analyzed hospitalized patients with Pilon fractures from January 2017 to December 2022 in a tertiary referral and university-affiliated hospital. Multivariate logistic regression analysis was used to identify risk factors for preoperative DVT, and a nomogram model was developed. Besides internal validation, patient data from January 2023 to December 2024 served as an external validation set to assess the model’s performance. A total of 1994 eligible patients were included, with 1432 in the training set and the others in the validation set. Multivariate analysis revealed 6 independent factors associated with preoperative DVT in patients with Pilon fractures. Risk factors (odds ratio [OR] > 1) included age >60 years (OR = 1.77, 95% confidence interval [CI]: 1.02–3.08, *P* = .044), delay from injury to duplex ultrasonography (days) (OR = 1.19, 95% CI: 1.13–1.26, *P* < .001), neutrophil-to-lymphocyte ratio > 3.17 (OR = 5.19, 95% CI: 2.70–9.98, *P* < .001), fasting blood glucose > 6.1 mmol/L (OR = 2.31, 95% CI: 1.08–4.95, *P* = .031), and D-dimer > 1.34 mg/L (OR = 3.96, 95% CI: 1.81–8.66, *P* = .001). Albumin was identified as a protective factor (OR = 0.87, 95% CI: 0.83–0.92, *P* < .001), indicating that low albumin levels correlate with increased DVT risk. The concordance index and Brier score of the nomogram were 0.829 and 0.033 in the training set, and the corrected values after internal validation were 0.796 and 0.035, respectively. The receiver operating characteristic curve, the calibration curve, the Hosmer–Lemeshow test, and the decision curve analysis performed well in both the training and validation cohorts. This study developed a personalized nomogram model with 6 predictors, which allows surgeons to stratify the risk of preoperative DVT in patients with Pilon fractures.

## 1. Introduction

Unlike common ankle fractures, Pilon fractures are typically caused by high-energy trauma, accounting for approximately 5% to 7% of all tibial fractures.^[1]^ Due to the increasing incidence of traffic accidents and falls, especially among the elderly population, the number of patients with Pilon fractures has been on the rise in recent years.^[2]^ Preoperative deep vein thrombosis (DVT) in patients with Pilon fractures, with an incidence of approximately 2.0% to 6.4%,^[3,4]^ not only induces or exacerbates swelling of the affected limb, disrupts local blood circulation, and impairs tissue healing, but also may spread proximally, leading to pulmonary embolism and even death. In addition, it has been reported that 75% to 100% of DVTs are often asymptomatic in the early stages,^[5]^ making it difficult to detect, and the treatment usually requires long-term anticoagulation therapy and repeated ultrasound examinations, leading to bleeding risk and high medical costs.^[6]^ Therefore, there is an urgent need to identify risk factors for preoperative DVT in patients with Pilon fractures, promising the potential for individualized assessment and intervention.

To our knowledge, current studies of post-traumatic DVT in orthopedics mainly focus on spinal fractures, hip fractures, and femur fractures, among other fracture types,^[7–9]^ with few studies focusing on DVT events in patients with Pilon fractures. Prophylactic anticoagulation for patients with Pilon fractures remains controversial. Although the American Orthopaedic Foot and Ankle Society and the American College of Chest Physicians explicitly note that active prophylaxis may benefit these patients with high-risk factors, such as a history of venous thromboembolism, specific risk factors have not been clearly outlined due to insufficient evidence.^[10]^ This ambiguity complicates clinical practice, as clinicians lack clear criteria to distinguish which patients truly need intervention. Even existing relevant research has limitations. Zhang et al conducted a retrospective analysis of 404 patients with lower extremity fractures, and the results showed that age was a significant risk factor for preoperative DVT.^[11]^ However, this small sample study primarily focused on other types of lower extremity fractures rather than specifically on Pilon fractures, and the variables included in the analysis were not comprehensive enough. In addition, due to the unique anatomical characteristics of Pilon fractures, such as thin soft tissue coverage of the distal tibia and high rates of soft tissue contusion, and the high-energy nature of the injuries, DVT predictive models developed for other fractures may not be directly applicable.^[12]^ In fact, developing a reliable preoperative DVT predictive model for Pilon fractures is critical. It can fill the gap in current guidelines regarding undefined risk factors, enabling clinicians to promptly identify high-risk individuals and implement targeted preventive measures.

Given this, this study aimed to determine the incidence and clinical risk factors for preoperative DVT in patients with Pilon fractures, while developing and validating a prediction nomogram.

## 2. Methods

### 2.1. General information

This retrospective study included all adult patients (aged 18 and above) who were admitted to our hospital due to Pilon fractures and received a standardized anticoagulation protocol from January 2017 to December 2024. The data from January 2017 to December 2022 were used for model development, while the data from January 2023 to December 2024 were further utilized for the external validation of this model. The data were accessed for research purposes on January 18, 2025. Exclusion criteria were: incomplete data, missing preoperative ultrasound, isolated intermuscular vein thrombosis, personal history of thrombotic diseases, anticoagulation and antiplatelet therapy within 3 months before admission, and multiple and old fractures. The study was approved by the Institutional Ethics Committee of the Third Hospital of Hebei Medical University, with approval number W20240211. The ethics committee waived the requirement for informed consent due to the nature of the retrospective, anonymized data analysis in this study. During the data collection period, the author did not have access to any personal information that could identify individual participants. Following the requirements for developing a clinical prediction model, the minimum sample size calculated with a target error of ≤0.05 is 470 cases. Therefore, our sample size (1432 cases) is sufficient.^[13]^

### 2.2. Data collection

The data analyzed for this study covered 4 aspects, including demographics, chronic comorbidities, injury-related data, and laboratory biomarkers. Demographic data included patients’ gender, age, body mass index, and living place. Comorbidities included alcohol history, smoking history, surgical history, allergy history, hypertension, heart disease, diabetes mellitus, cerebrovascular disease, hyperlipidemia, liver disease, kidney disease, autoimmune diseases, peripheral vascular disease, and tumors. Injury-related data included the mechanism of injury, fracture blisters, open fractures, fracture classification according to the Arbeitsgemeinschaft für Osteosynthesefragen/Orthopaedic Trauma Association classification system, Tscherne classification,^[14]^ time from injury to admission, and time from injury to duplex ultrasonography (DUS) examination. Laboratory biomarkers included red blood cells, hemoglobin, white blood cells, neutrophils, lymphocytes, neutrophil-to-lymphocyte ratio (NLR), and platelets; levels of fasting blood glucose (FBG), total cholesterol, triglycerides, high-density lipoprotein cholesterol, low-density lipoprotein cholesterol, very low-density lipoprotein, total protein, albumin (ALB), globulin, high-sensitivity C-reactive protein (HCRP), antithrombin III, prothrombin time, fibrinogen, thrombin time and activated partial thromboplastin time, and D-dimer. If the patient had multiple hematology tests before the diagnosis of DVT, the most recent test results were selected.

### 2.3. Detection and management of DVT

Within 24 hours of admission, all participants underwent fasting venous blood sampling. The DUS examination of both lower extremities was typically performed within 24 to 48 hours after admission, and follow-up scans were conducted every 3 days. DVT was detected based on signs of deep venous lumen obstruction or filling defects. The involved veins included the common femoral vein, superficial femoral vein, deep femoral vein, popliteal vein, posterior tibial vein, anterior tibial vein, and peroneal vein. Thrombosis of the superficial or intermuscular veins of the lower extremities was excluded due to its relatively minor clinical significance. At least 2 ultrasound specialists confirmed the diagnosis of DVT. All patients included in this study received a standardized preoperative thromboprophylaxis regimen consisting of both non-pharmacological and pharmacological measures. Non-pharmacological prophylaxis was initiated within 6 hours of admission and included elevating the affected limb to 30° above heart level to reduce venous stasis, encouraging a daily oral water intake of at least 2000 mL to avoid hemoconcentration, guiding passive and active range-of-motion exercises of the unaffected lower limb (e.g., ankle pumps and straight-leg-raising exercises) 3 to 4 times per day, and applying intermittent pneumatic compression devices to both lower limbs for 30 minutes per session, twice daily, to promote venous return. Within 24 hours after admission, all patients included in this study received pharmacological prophylaxis with low-molecular-weight heparin (LMWH; e.g., enoxaparin sodium 4000 AxaIU subcutaneously once daily), and the regimen was subsequently adjusted according to DUS findings: patients without DVT continued prophylactic-dose LMWH, whereas patients with DVT received therapeutic-dose LMWH (e.g., enoxaparin sodium 6000 AxaIU subcutaneously twice daily) to prevent thrombus progression; LMWH medication was discontinued at least 12 hours before the operation.^[15]^

### 2.4. Statistical analysis

Continuous variables were presented as mean ± standard deviation or median [Q1–Q3], based on the normal or skewed distribution, and compared using Student *t* test or Mann–Whitney *U* test, as appropriate. Categorical variables were expressed as numbers (%) and analyzed with the chi-square or Fisher exact test. These tests were used for univariate screening, involving direct group comparisons between DVT and non-DVT patients for all candidate variables prior to multivariable modeling. Plasma D-dimer is a valuable indicator for the diagnosis of DVT, but it faces the limitation of poor specificity. Therefore, in this study, the Youden index was applied to determine the optimal cutoff value.^[16]^ The same strategy was adopted for NLR and HCRP.

Variables with two-sided *P* < .05 in the aforementioned univariate group comparisons were included in the multivariate stepwise backward logistic regression analysis to determine the independent risk factors for preoperative DVT in patients with Pilon fractures. The variance inflation factor (VIF) was used to test for multicollinearity. The “rms” and “DynNom” packages in R software were used to develop the simple and web-based nomogram model. To evaluate its discriminative power, the receiver operating characteristic (ROC) curve was employed, presenting the area under the curve (AUC) and concordance index (C-index). Superior discriminatory performance is reflected by larger AUC and C-index values, nearing 1.0.^[17,18]^ The calibration curve was used to evaluate the accuracy of the absolute risk prediction value of the model and was further evaluated by the Hosmer–Lemeshow goodness-of-fit test. The Brier score can be interpreted as an extension of the Hosmer–Lemeshow test; the value closer to 0, the better the model calibration. Decision curve analysis (DCA) was carried out to assess the nomogram’s clinical utility, estimating net clinical benefit across diverse threshold probabilities.^[19]^ For internal validation, the bootstrap resampling method (1000 repetitions) was applied to acquire corrected C-index and Brier score estimates. External validation was also executed using an independent dataset to examine model generalizability.

All statistical analyses were implemented with R software (version 4.1.3, Foundation for Statistical Computing, Vienna, Austria), and statistical significance was set at a two-sided *P* value <0.05.

## 3. Results

### 3.1. Demographic data

**Figure 1.**
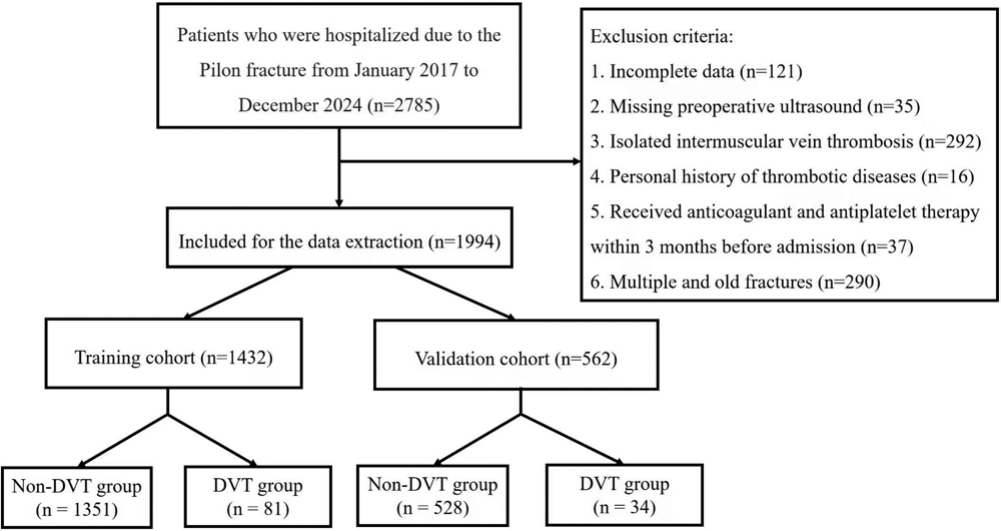
Patient selection flowchart. DVT = deep vein thrombosis.

According to the inclusion and exclusion criteria of this study, we selected 1994 individuals from 2785 patients hospitalized for Pilon fracture as the study population, of which 1432 were used as the model development cohort (Fig. 1). In the development dataset, 1039 (72.56%) were males and 393 (27.44%) were females; the mean age was (43.8 ± 11.0) years, and 17.7% (254/1432) of the patients were older than 60 years. Of these patients, 81 (5.66%, 81/1432) were identified with lower extremity DVT. 4 (4.94%, 4/81) DVTs involved the common femoral vein, 9 (11.11%, 9/81) involved the popliteal vein, 55 (67.90%, 55/81) involved the anterior or posterior tibial vein, and 13 (16.05%, 13/81) involved the peroneal vein.

### 3.2. Univariate and multivariate regression analyses

By calculating the maximum Youden index, the new cutoff values identified in this study are 1.34 mg/L for D-dimer, 3.17 for the NLR, and 27.52 mg/L for HCRP. A total of 16 potential predictors were identified by the unifactorial analysis (Table 1). In the collinearity assessment, when “time from injury to admission” and “time from injury to DUS” were entered simultaneously into the multivariable logistic regression model, their VIFs were 11.3 and 12.1, respectively, indicating substantial multicollinearity. Given the severe collinearity, only one of the 2 time variables was retained. “Time from injury to DUS” was selected for the final model, with “time from injury to admission” consequently excluded. The multifactorial logistic regression results showed that advanced age, delay from injury to DUS, NLR > 3.17, high fasting glucose, low level of ALB, and D-dimer > 1.34 mg/L were independent risk factors for preoperative DVT in patients with Pilon fracture (Table 2). All the variables included in the multivariate regression had a VIF value <10 (max VIF: 1.2 [NLR]), indicating no significant multicollinearity.

**Table 1:** Univariate analysis of baseline variables of interest between DVT and non-DVT patients.

Variables	Non-DVT patients (n = 1351)	DVT patients (n = 81)	*P* value
Gender (female)	374 (27.7%)	19 (21.6%)	.331
Age	43.6 ± 10.9	47.8 ± 11.2	.001*
>60 yr	231 (17.1%)	23 (28.4%)	.010*
BMI (kg/m^2^)	24.2 ± 2.6	25.7 ± 0.9	.089
Living place (rural)	390 (28.9%)	18 (22.2%)	.198
Alcohol history			.909
No	442 (32.72)	27 (33.33)	
Yes	909 (67.28)	54 (66.67)	
Smoking history			.011*
No	904 (66.9%)	43 (53.1%)	
Yes	447 (33.1%)	38 (46.9%)	
Surgical history	61 (4.5%)	7 (8.6%)	.090
Allergy history	127 (9.4%)	10 (12.3%)	.381
Hypertension	111 (8.2%)	9 (11.1%)	.361
Diabetes mellitus	105 (7.8%)	11 (13.6%)	.063
Heart disease	45 (3.3%)	4 (4.9%)	.647
Cerebrovascular disease	36 (2.7%)	3 (3.7%)	.836
Hyperlipidemia	119 (8.8%)	14 (17.3%)	.011*
Liver disease	49 (3.6%)	6 (7.4%)	.155
Kidney disease	42 (3.1%)	5 (6.2%)	.237
Autoimmune diseases	39 (2.9%)	3 (3.7%)	.933
Peripheral vascular disease	45 (2.3%)	6 (7.4%)	.106
Tumors	14 (1.0%)	3 (3.7%)	.104
Fracture blister	735 (54.4%)	53 (65.4%)	.053
Open fracture	653 (48.3%)	46 (56.8%)	.139
Mechanism of injury			.500
Flat land tumble	459 (33.97)	23 (28.40)	
Traffic injury	479 (35.46)	29 (35.80)	
High falling	413 (30.57)	29 (35.80)	
Fracture classification			.226
A	280 (20.73)	22 (27.16)	
B	603 (44.63)	29 (35.80)	
C	468 (34.64)	30 (37.04)	
Tscherne			.076
0	220 (16.28)	9 (11.11)	
1	610 (45.15)	32 (39.51)	
2	335 (24.80)	21 (25.93)	
3	186 (13.77)	19 (23.46)	
Time from injury to admission (days)	1.0 (1.0, 3.0)	4.0 (2.0, 7.0)	<.001*
Time from injury to DUS (days)	2.0 (1.0, 5.0)	4.0 (2.0, 9.0)	<.001*
RBC < lower limitation	736 (54.5%)	54 (66.7%)	.032*
HGB < lower limitation	528 (39.1%)	42 (51.9%)	.023*
WBC > 9.5 × 10^9^/L	334 (24.7%)	24 (29.6%)	.322
NEU (> 6.3 × 10^9^/L)	424 (31.4%)	33 (40.7%)	.079
LYM (< 1.8 × 10^9^/L)	762 (56.4%)	55 (67.9%)	.042*
NLR > 3.17	288 (21.3%)	50 (61.7%)	<.001*
PLT > 300 × 10^9^/L	124 (9.2%)	10 (12.3%)	.342
FBG > 6.1 mmol/L	199 (14.7%)	30 (37.0%)	<.001*
TC > 5.8 mmol/L	105 (7.8%)	11 (13.6%)	.063
TG > 1.7 mmol/L	150 (11.1%)	13 (16.0%)	.173
HDL-C < 0.8 mmol/L	472 (34.9%)	37 (45.7%)	.050
LDL-C > 3.1 mmol/L	217 (16.1%)	21 (25.9%)	.021*
VLDL > 0.8 mmol/L	193 (14.3%)	14 (17.3%)	.456
TP (g/L)	62.6 ± 5.4	60.8 ± 3.6	.081
ALB (g/L)	36.2 ± 4.9	33.4 ± 4.8	<.001*
GLOB (g/L)	24.4 ± 3.7	35.6 ± 3.9	.314
HCRP > 27.52 mg/L	319 (23.6%)	28 (34.6%)	.025*
PT (>12.5 s)	287 (21.2%)	22 (27.2%)	.209
FIB > 4 g/L	357 (26.4%)	33 (40.7%)	.005*
TT < 12 s	153 (11.3%)	14 (17.3%)	.105
APTT < 28 s	298 (22.1%)	24 (29.6%)	.113
D-dimer > 1.34 mg/L	270 (19.99)	35 (43.2)	<.001*

Reference range: female, 3.5–5.0 × 10^12^/L; males, 4.0–5.5 × 10^12^/L.

Reference range: females, 110–150 g/L; males, 120–160 g/L.

APTT = activated partial thromboplastin time, BMI = body mass index, DVT = deep vein thrombosis, FIB = fibrinogen, GLOB = globulin, HCRP = high-sensitivity C-reactive protein, HDL-C = high-density lipoprotein cholesterol, HGB = hemoglobin, LDL-C = low-density lipoprotein cholesterol, LYM = lymphocytes, NEU = neutrophil, PLT = platelet, RBC = red blood cell, TC = total cholesterol, TG = triglyceride, TP = total protein, TT = thrombin time, VLDL = very low-density lipoprotein, WBC = white blood cell.

* Statistical significance.

**Table 2:** Multivariate analyses of the independent risk factors associated with preoperative DVT.

Variables	*P* value	Odds ratio	95% CI
Delay from injury to DUS (days)	<.001	1.19	1.13–1.26
Age > 60 yr	.044	1.77	1.02–3.08
NLR > 3.17	<.001	5.19	2.70–9.98
FBG > 6.1 mmol/L	.031	2.31	1.08–4.95
ALB	<.001	0.872	0.83–0.92
D-dimer (>1.34 mg/L)	.001	3.96	1.81–8.66

ALB = albumin, CI = confidence interval, DUS = duplex ultrasonography, DVT = deep vein thrombosis, FBG = fasting blood glucose, NLR = neutrophil-to-lymphocyte ratio.

### 3.3. Development and validation of a nomogram model

**Figure 2.**
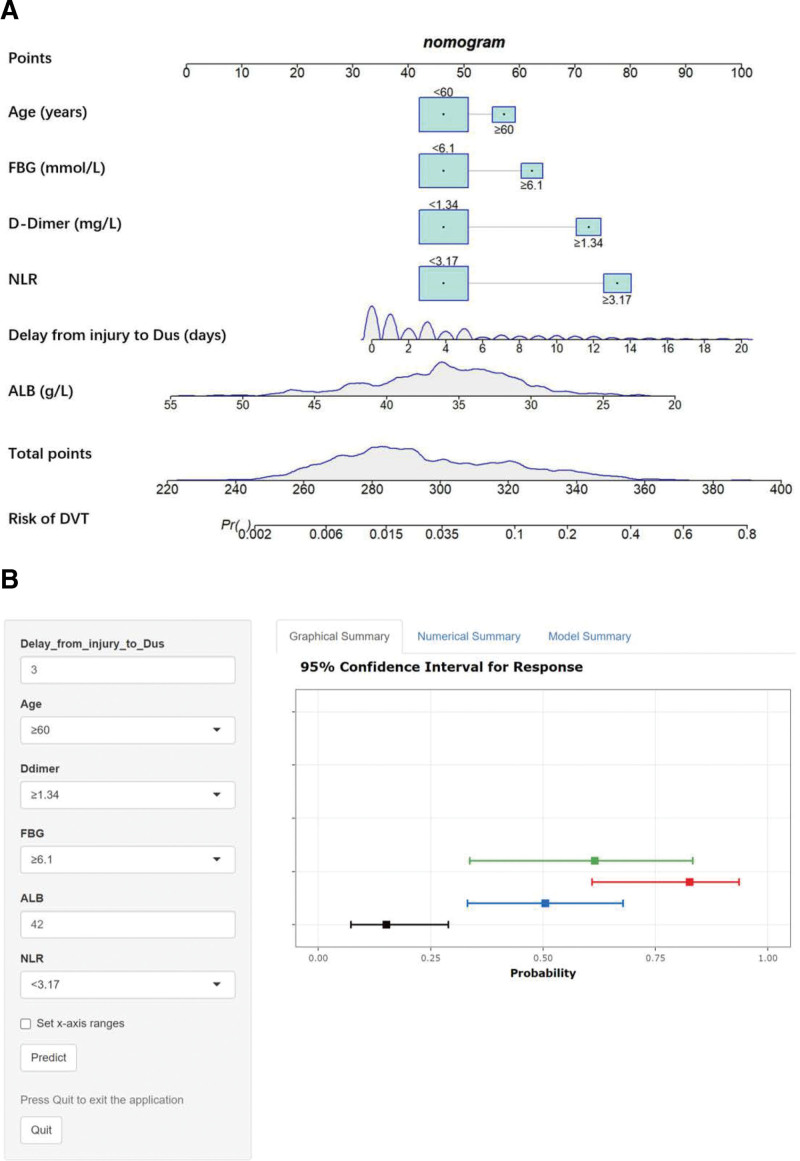
The simple nomogram (A) and web-based nomogram (B) for predicting the risk of preoperative DVT in patients with Pilon fractures. Points are assigned for each variable, including age, D-dimer, FBG, NLR, delay from injury to DUS, and ALB. The total points correspond to the risk of DVT (access to online nomogram: https://dynanomogram.shinyapps.io/dynnomapp-1/). ALB = albumin, DVT = deep vein thrombosis, FBG = fasting blood glucose, NLR = neutrophil-to-lymphocyte ratio.

**Figure 3.**
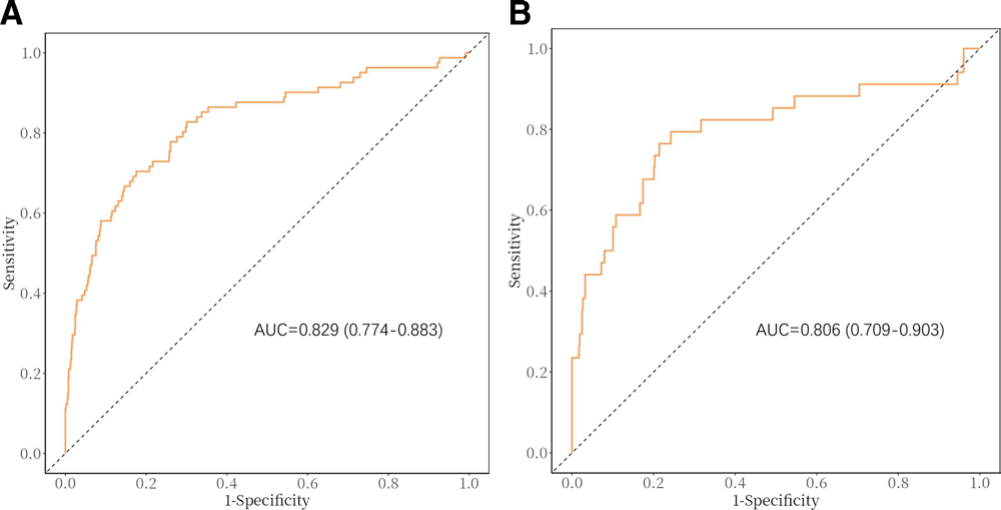
Receiver operating characteristic (ROC) curves of the nomogram model in the training set (A) and validation set (B). The AUC of the nomogram in the training and validation sets was 0.829 and 0.806, respectively, indicating that the model had good discrimination ability. AUC = area under the curve, ROC = receiver operating characteristic.

**Figure 4.**
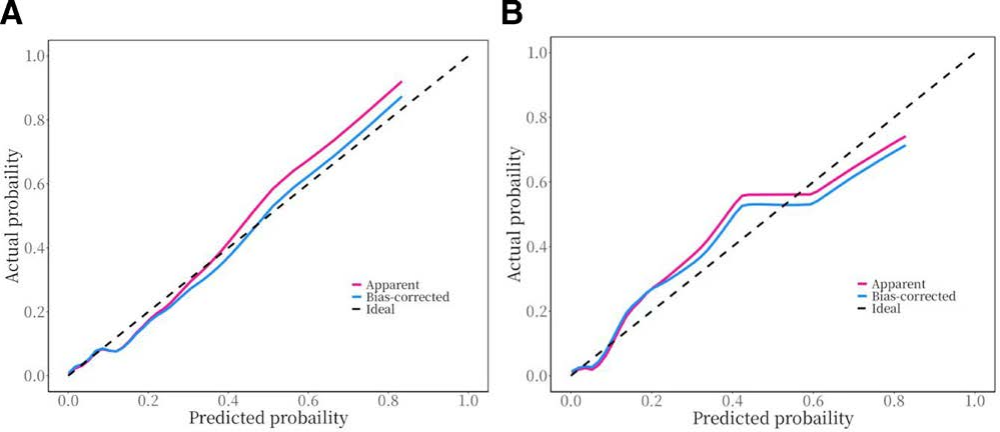
Calibration curves of the nomogram in the training set (A) and validation set (B). The x-axis represents the predicted probability of DVT, and the y-axis represents the actual probability of DVT. The diagonal dashed line represents a perfect prediction by an ideal model. The closer the red and blue curves are to the ideal dashed line, the better the predictive accuracy of the nomogram. DVT = deep vein thrombosis.

A nomogram model was developed based on the logistic regression results. The simple nomogram (Fig. 2A) can be used without restriction. When applying the model, the patient’s age is 1st placed on the axis of the specified variable. A straight line is then drawn up the axis at that point to determine the risk score. Each variable is repeated, and a total score is calculated. The predicted probability of preoperative DVT risk in a patient with a Pilon fracture is identified by finding the final sum on the “total score” axis and then drawing a vertical line to intersect the predicted probability axis. The web-based nomogram (Fig. 2B) allows surgeons to input the results of each covariate while connected to the network, displaying the predicted probability of DVT with a 95% confidence interval (CI). The model had an AUC of 0.829 (95% CI: 0.774–0.883, Fig. 3), with high sensitivity and specificity (82.7% and 72.6%), indicating good predictive ability. The C-index and Brier score were 0.829 and 0.033, respectively. According to bootstrap internal validation (1000 replications), the calibrated values were 0.796 and 0.035, respectively, which indicates the overall satisfactory performance of the model. The Hosmer–Lemeshow χ^2^ statistics for the calibration curve (Fig. 4) in the training and testing datasets were 8.572 (*P* = .381) and 13.115 (*P* = .179), respectively. These results indicate good consistency between the predicted probability of preoperative DVT and the actual occurrence probability in patients with Pilon fractures. More importantly, the model performed equally well in the external validation cohort with an AUC of 0.806.

**Figure 5.**
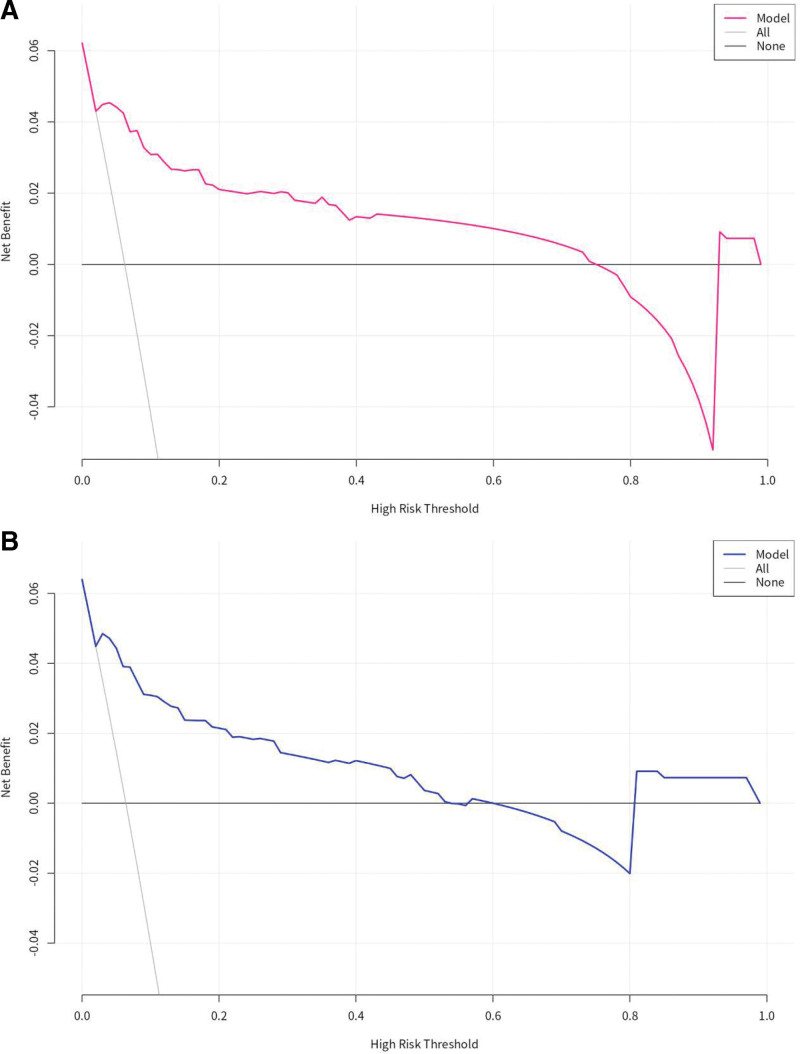
Decision curve analysis (DCA) was performed on the nomogram in the training set (A) and validation set (B). DCA showed that the net benefit of the training model was higher in the threshold probability interval of 2% to 74%, and the net benefit of the validation model was higher in the threshold probability interval of 2% to 52%. DCA = decision curve analysis.

In addition, we performed a DCA on the predictive model. The results (Fig. 5) showed that the model improved the net benefit of the “treat all” or “no treatment” scenarios when the threshold probability was between 2% and 81%. DCA performance on the validation dataset also showed that the model performed well in guiding clinical practice.

## 4. Discussion

In this study, the incidence of preoperative DVT in patients with a Pilon fracture was 5.66%. Advanced age, delay from injury to DUS, NLR > 3.17, high fasting glucose, low level of ALB, and D-dimer > 1.34 mg/L were identified as independently associated with high risk of preoperative DVT. A nomogram model containing 6 high-value predictors was constructed with a sensitivity of 75.8%, a specificity of 83.7%, and an AUC value of 0.829 (95% CI: 0.774–0.883). More importantly, the model still showed favorable DVT prediction performance after bootstrap 1000 replicate sampling validation and external validation.

The preoperative DVT incidence in our Pilon fracture cohort (5.66%) is highly comparable to the 6.4% reported by Luo et al for lower-extremity DVT following ankle fractures.^[4]^ Moreover, the key risk factors are consistent across the 2 studies: older age, low ALB level, elevated D-dimer, and lymphocyte-based inflammatory dysregulation (captured in our analysis by the NLR), despite differences in fracture pattern and cohort composition. The consistent incidence and core risk factors across studies in similar populations provide additional support for the robustness and external applicability of our model.

In this study, we found a 77% increased risk of preoperative DVT in elderly Pilon fracture patients, which is similar to different studies in numerous fields.^[20–22]^ Zhang et al noted that age is a key risk factor for preoperative, postoperative, and 1-month postoperative DVT in patients with lower extremity fractures.^[11]^ Advanced age can lead to loss of functional tissue cells and reduced capacity for self-renewal and maintenance, resulting in vascular aging characterized by progressive smooth muscle cell and endothelial dysfunction.^[23]^ Vessel wall thickening, reduced elasticity, and slower blood flow caused by these factors are directly related to an increased risk of DVT.^[24]^ In addition, older adults often suffer from underlying conditions such as hypertension and hyperlipidemia, which can further damage the vascular endothelium and activate the coagulation system, thereby increasing the risk of DVT in patients with Pilon fractures.^[25]^ Therefore, priority attention should be given to elderly patients, and more aggressive DVT prophylaxis should be recommended.

D-dimer is a specific fibrin degradation product generated from cross-linked fibrin under the action of plasmin, and it is the most important laboratory biomarker reflecting thrombus formation and thrombolytic activity.^[26]^ As the simplest fibrin degradation product, its concentration can vary with numerous factors such as thrombosis, trauma, and infection.^[27]^ This implies that D-dimer has a relatively low specificity in the diagnosis or prediction of DVT.^[28]^ Given this, this study used the method of the maximum Youden index to redefine the optimal cutoff value of D-dimer as 1.34 mg/L. Compared with the traditional threshold level of 0.5 mg/L, the adjusted value significantly increased the specificity from 29.8% to 70.2%. Moreover, the decreased sensitivity can ultimately be compensated for by the fitting of the model, thereby improving the accuracy of prediction.

High FBG levels and low ALB levels are recognized risk factors for postoperative DVT in surgical patients.^[29–31]^ Hyperglycemia can cause damage to vascular endothelial cells and trigger an inflammatory response, having a widespread impact on systemic vascular sclerosis. One of the adverse consequences of these factors is thrombosis.^[32]^ In addition, hyperglycemia promotes platelet activation, increasing the body’s coagulation activity. Moreover, long-term hyperglycemia leads to the formation of a large amount of glycated hemoglobin, which increases the rigidity of red blood cells and significantly raises the blood viscosity accordingly.^[33]^ A decrease in ALB levels reduces plasma colloid osmotic pressure, increases vascular permeability, leads to more exudate, increases the proportion of blood cells, and further elevates blood viscosity. With the extravascular leakage of blood cells and antithrombin, the level of antithrombin in the plasma decreases, further exacerbating the risk of thrombosis.^[34]^ These factors mutually promote and influence each other, contributing to the development of DVT. The results of this study suggest that blood glucose and ALB can be regulated as nutritional or metabolic markers, and active correction of these markers has great clinical potential to prevent thrombosis and improve prognosis.

The NLR reflects the state of systemic inflammation and immune response, and its potential association and predictive value with perioperative DVT events in patients undergoing major orthopedic surgery has been confirmed by studies.^[35,36]^ This may be because both the neutrophil count and the lymphocyte count reached the critical values of statistical significance in the univariate analysis, and the ratio of the 2 further amplifies this predictive effect. Recently, Gao et al, through a retrospective analysis of the clinical data of 1103 patients with ankle fractures, found that the preoperative NLR levels were significantly higher in patients with DVT.^[37]^ The underlying mechanism may be related to the release of neutrophil extracellular traps after the activation of neutrophils, as well as the damage to the vascular endothelium by pro-inflammatory cytokines such as interleukin-6. These factors can activate the platelet and coagulation pathway.^[38]^ Meanwhile, a decrease in lymphocytes can weaken immune regulation, leading to the continuous release of neutrophil extracellular traps and insufficient secretion of interleukin-10, which results in the inability to antagonize pro-inflammatory cytokines and ultimately increases the risk of DVT.^[39]^ The results indicate that NLR can be incorporated as a novel high-value inflammatory indicator into the development of predictive models.

In this study, we found that Pilon fracture patients with DVT had a significantly longer waiting time for DUS examination than those without DVT. Multivariate analysis further showed that for each additional day of delay from trauma onset to DUS performance after admission, the risk of DVT increased by 19%. In this study, the long wait time for DUS examination was mainly attributed to the delay from injury to admission, which is very common in large tertiary hospitals with high numbers of patients referred from remote hospitals, especially for patients with multiple injuries. In addition, due to the special circumstances of patients with Pilon fractures, patients with limb swelling often have to wait 1 to 2 weeks for the swelling to disappear before surgical intervention can be performed to reduce the incidence of wound complications.^[40]^ Based on the results of this study, we recommend that patients with Pilon fractures who have delayed admission receive DUS as early as possible, and that anticoagulation and enhanced preoperative management be performed if necessary to minimize the risk of DVT.

It is worth noting that both “time from injury to admission” and “time from injury to DUS” were significant in the univariable analysis. Therefore, it is necessary to discuss why the former was excluded from the multivariate modeling. When both time variables were entered into the same multivariable model, their VIFs were 11.3 and 12.1, respectively, while VIFs for the remaining predictors were all <2, indicating substantial multicollinearity confined to these 2 measures. This issue would inflate standard errors and yield unstable coefficient estimates, undermining interpretability and model stability. Therefore, we prioritized the variable with greater clinical relevance and better predictive performance. “Time from injury to DUS” directly reflects the interval between trauma and gold-standard DVT screening, so its delay prolongs thrombosis risk and allows targeted intervention (e.g., prioritizing DUS for delayed-admission patients); in contrast, “time from injury to admission” is an indirect factor with limited clinical modifiability. Further preliminary model analysis confirmed that the model incorporating “time from injury to DUS examination” had superior predictive performance (C-index = 0.829, Brier score = 0.033), better than the model with “time from injury to admission” (C-index = 0.805, Brier score = 0.043). Selecting “time from injury to DUS” resolved collinearity, retained clinical actionability, and boosted the model’s preoperative DVT risk stratification performance in Pilon fractures.

Nomogram prediction models rely on multi-factor analysis methods, integrating multiple predictive factors to achieve personalized and accurate prediction of the probability of outcome events.^[41]^ A highlight of this study is that it explores the risk factors for preoperative DVT in patients with Pilon fractures by expanding the sample size and successfully constructs a prediction model. The 6 predictive factors included in this model can be obtained shortly after the patient’s admission. Using this intuitive and concise model, the total risk assessment score can be calculated, and then the predicted probability of preoperative DVT can be accurately obtained, providing strong support for clinical early intervention.

Any study has its limitations. First, the study inherently carries unavoidable selection bias due to its retrospective nature. Second, to improve the accuracy of the prediction model, we limited the analysis to adults and excluded patients with incomplete data, missing preoperative ultrasound, isolated intermuscular vein thrombosis, a prior history of thrombotic disease, anticoagulation or antiplatelet therapy within 3 months before admission, or multiple/old fractures. Therefore, this model may not be applicable to children and these excluded patient groups. Third, the model was constructed based on the static data of patients at admission, failing to consider the impact of dynamic factors such as disease progression and treatment interventions from admission to pre-operation on the risk of DVT. Fourth, although regression analysis was employed, unrecognized or difficult-to-measure confounding factors, such as information on medication use before admission, were not effectively controlled or analyzed in this study. Fifth, the nomogram was derived from a single-center cohort of patients with Pilon fractures who were managed with a standardized LMWH-based pharmacologic and mechanical thromboprophylaxis regimen. While this strategy is within the range of guideline-recommended options for orthopedic trauma, it differs from other commonly used regimens, such as aspirin-based antiplatelet protocols or prophylaxis with direct oral anticoagulants (e.g., rivaroxaban or apixaban).^[6]^ Therefore, the absolute risks predicted by the model should be extrapolated cautiously to patients receiving other prophylaxis strategies, and further validation in such settings is warranted.

## 5. Conclusion

The incidence of preoperative DVT in patients with Pilon fractures was 5.66%. We constructed a prediction nomogram model based on 6 independent predictive factors, including advanced age, delay from injury to DUS examination, NLR, FBG, ALB, and D-dimer. The model performed well in both internal and external validation. Surgical teams should recognize the importance of actively optimizing these risk factors to reduce the incidence of DVT and minimize the possibility of catastrophic medical consequences.

## Acknowledgments

We sincerely thank all the patients for their participation in this study. This work was supported by the Hebei Province Medical Science Research Project Plan (No. 20241236). The authors declare that they have no conflicts of interest.

## Author contributions

**Conceptualization:** Tong Li, Xuebin Zhang, Chenni Ji.

**Methodology:** Yongxuan Kang, Chenni Ji.

**Data curation:** Yongxuan Kang, Kai Kang.

**Formal analysis:** Yang Zhang.

**Writing – original draft:** Yongxuan Kang, Chenni Ji.

**Writing – review and editing**: Yongxuan Kang, Yang Zhang, Kai Kang, Tong Li, Xuebin Zhang, Chenni Ji.
